# Genome-Wide Association Study of Exercise Addiction Among Elite Wrestlers

**DOI:** 10.3390/brainsci15020102

**Published:** 2025-01-22

**Authors:** Celal Bulgay, Anıl Kasakolu, Türker Bıyıklı, Seyrani Koncagul, Hasan H. Kazan, Ildus I. Ahmetov, Mehmet A. Ergun, Mark D. Griffiths, Attila Szabo

**Affiliations:** 1 Sports Science Faculty, Bingol University, Bingol 12000, Türkiye; cbulgay@bingol.edu.tr; 2 Graduate School of Natural and Applied Sciences, Ankara University, Ankara 06135, Türkiye; kasakoluanil1@gmail.com; 3 Sports Science Faculty, Marmara University, İstanbul 34722, Türkiye; turker.biyikli@marmara.edu.tr; 4 Department of Animal Science, Faculty of Agriculture, Ankara University, Ankara 06135, Türkiye; seyranikoncagul@gmail.com; 5 Department of Medical Biology, Gulhane Faculty of Medicine, University of Health Sciences, Ankara 06010, Türkiye; hasanhuseyinkazan@gmail.com; 6 Laboratory of Genetics of Aging and Longevity, Kazan State Medical University, 420012 Kazan, Russia; genoterra@mail.ru; 7 Research Institute for Sport and Exercise Sciences, Liverpool John Moores University, Liverpool L3 3AF, UK; 8 Department of Medical Genetics, Faculty of Medicine, Gazi University, Ankara 06560, Türkiye; aliergun@gazi.edu.tr; 9 International Gaming Research Unit, Psychology Department, Nottingham Trent University, Nottingham NG1 4FQ, UK; mark.griffiths@ntu.ac.uk; 10 Faculty of Health and Sport Sciences, Széchenyi István University, H-9026 Győr, Hungary

**Keywords:** DNA, polymorphism, genotype, GWAS, genetic predisposition, exercise addiction, physical activity, behavior, athletes, wrestling

## Abstract

Background: Exercise addiction, marked by an inability to control exercise and associated with distress that clinically impairs daily activities, is a significant but underrecognized issue in physical activity and health. While its physiological, psychological, and behavioral aspects have been studied, the genetic basis of exercise addiction remains poorly understood, requiring further investigation. The present study conducted a genome-wide association study of exercise addiction among elite Turkish wrestlers. Methods: The sample comprised 67 male wrestlers (34 freestyle wrestlers and 33 Greco-Roman wrestlers). Exercise addiction was assessed using the Exercise Addiction Scale. Whole-genome genotyping was performed using DNA microarray. Results: Using a genome-wide approach (*p* < 1.0 × 10^−^⁵), we identified six suggestively significant single-nucleotide polymorphisms (SNPs) associated with exercise addiction status. Of these, the high-addiction alleles of five SNPs (*PRDM10* rs74345126, near *PTPRU* rs72652685, *HADHB* rs6745226, *XIRP2* rs17614860, and near *GAREM2* rs1025542) have previously been associated with an increased risk of mental health disorders such as anxiety and depression or higher levels of physical activity. We also examined potential associations between the genetic markers previously linked to addiction-related traits such as obsessive–compulsive disorder and cigarette smoking, and personality traits linked to negative emotions including neuroticism. Using this candidate gene approach (*p* < 0.05), we identified three additional SNPs associated with exercise addiction in the same direction of association (*DEFB135* rs4841662, *BCL11A* rs7599488, and *CSRNP3* rs1551336). Conclusions: The present study provides preliminary evidence for the genetic basis of exercise addiction, highlighting specific SNPs that may play a role in the development of this condition among elite wrestlers.

## 1. Introduction

Exercise is defined as a collective, planned, and repetitive form of physical activity designed to enhance physical capacity and improve overall performance [1]. Regular exercise offers numerous benefits across the physical, psychological, and social domains. These benefits include improved cardiovascular health and muscle strength, weight management, stress reduction, enhanced mood, and strengthened social bonds. However, when exercise is practiced excessively and without regulation, it can lead to a maladaptive condition widely known as exercise addiction [2,3,4].

Exercise addiction is a behavioral condition characterized by the loss of control over regulating exercise routines. Individuals affected by this condition exhibit compulsive patterns of behavior, progressively increasing the duration, frequency, and intensity of their exercise to achieve desired physiological or psychological effects. This compulsive behavior often takes precedence over other important aspects of life, including social relationships, professional responsibilities, and personal well-being, ultimately leading to clinically significant impairments in daily functioning [5,6,7].

Those suffering from exercise addiction frequently experience both physiological and psychological distress when they are unable to engage in their routines. This distress can manifest in various forms, such as chronic injuries caused by overtraining, social isolation due to prioritizing exercise over relationships, and mental health issues such as anxiety and depression [8,9,10]. In severe cases, the detrimental effects of exercise addiction may extend to disruptions in an individual’s professional and personal life, further exacerbating the individual’s overall well-being.

Despite its well-documented negative consequences, exercise addiction remains an under-recognized issue within mental health literature. While it is widely acknowledged as a maladaptive condition that can significantly impair physical, psychological, and social well-being, the absence of specific diagnostic criteria and its non-inclusion in the fifth edition of the American Psychiatric Association’s *Diagnostic and Statistical Manual of Mental Disorders* (DSM-5 [11]) has hindered its formal classification as a distinct mental health disorder. This omission creates several challenges for healthcare professionals and researchers. Without standardized diagnostic criteria, it is difficult to accurately identify and differentiate exercise addiction from other conditions, such as obsessive–compulsive disorder, eating disorders, or general fitness enthusiasm [11,12,13].

Many cases may go undiagnosed or be misdiagnosed, delaying the necessary interventions. Moreover, the lack of formal recognition limits the development of targeted treatment protocols and evidence-based interventions specifically tailored to address exercise addiction. Individuals struggling with this condition often face significant barriers to care because healthcare systems may not recognize the severity of their symptoms or provide adequate resources for treatment. Additionally, social perceptions further obscure the issue. Exercise is frequently celebrated as a positive and virtuous activity, making it harder to distinguish when it becomes compulsive and harmful. To address these challenges, increased research, awareness, and the establishment of diagnostic criteria are essential. Recognizing exercise addiction as a distinct disorder would not only validate the experiences of the affected individuals but also pave the way for effective prevention and treatment strategies, improving overall mental healthcare [2,4,14].

The underlying causes of exercise addiction have received increasing attention in recent years, with a growing body of research identifying a multifactorial etiology involving psychological, behavioral, and motivational dimensions. Psychological traits such as perfectionism, obsessive–compulsive tendencies, neuroticism, low self-esteem, and sensitivity to reward and punishment, are frequently observed among individuals prone to exercise addiction [15]. Behavioral patterns, including the progressive escalation of exercise duration and intensity, rigid adherence to routines, and the prioritization of exercise over other responsibilities, further contribute to the development of compulsive exercise habits [9]. Additionally, social expectations and cultural pressures tied to physical activity such as the idealization of specific body types or the perception of exercise as a marker of discipline and success can exacerbate the risk of developing addiction-like behaviors [16,17]. Motivational factors, both intrinsic (e.g., personal satisfaction and mastery) and extrinsic (e.g., external validation and avoidance of guilt), also play a critical role in sustaining these behaviors [18].

Recently, the exploration of genetic factors has provided new insights into the complexity of exercise addiction. This emerging field, known as sport psychogenetics, seeks to integrate genetic and psychological perspectives to better understand the biological and environmental interplay underlying exercise addiction [19]. Several candidate genes have been identified, linking genetic predispositions to psychological traits among athletic populations [20,21,22,23]. Notable genes include dopamine receptor D2 (*DRD2*) and catechol-O-methyltransferase (*COMT*), both of which influence dopamine signaling and are associated with reward sensitivity and compulsive behaviors. Brain-derived neurotrophic factor (*BDNF*), known for its role in neuroplasticity and stress response, has been implicated in mood regulation and behavioral persistence. Similarly, 5-hydroxytryptamine receptor 1A (*HTR1A*) and cholinergic receptor muscarinic 2 (*CHRM2*) have been associated with mood stabilization and emotional regulation, traits that may influence exercise behaviors. Other behavior-related genes are gamma-aminobutyric acid type A receptor subunit alpha 6 (*GABRA6*), which plays a role in inhibitory neurotransmission and anxiety regulation, and oxytocin receptor (*OXTR*), which has been linked to social bonding and stress coping mechanisms. Leucine-rich repeat neuronal 3 (*LRRN3*), involved in neuronal development, and ankyrin repeat and kinase domain containing 1 (*ANKK1*), affecting dopamine signaling, have also been studied for their behavioral and psychological impacts among athletes [20,21,23,24,25,26,27].

In sport psychogenetics, of particular interest is the *ANKK1* gene due to its potential association with exercise addiction. Variations in this gene, particularly those influencing dopamine receptor function, may predispose individuals to reward-seeking and compulsive behaviors, both of which are central to the development of exercise addiction [28,29]. Research suggests that *ANKK1* polymorphisms may heighten sensitivity to the rewarding aspects of exercise, leading to patterns of behavior that resemble addiction. This aligns with broader findings in psychogenetics, where the genes influencing the dopamine pathways often play critical roles in addictive and compulsive behaviors. The genetic basis of exercise addiction has predominantly been explored through studies focusing on specific polymorphisms, particularly within the *ANKK1* gene. These studies have provided valuable insights into the potential role of the dopamine pathways in shaping compulsive exercise behaviors. However, single-gene approaches inherently limit the ability to capture the broader and complex genetic architecture underlying exercise addiction. This condition, like many other behavioral phenotypes, is likely influenced by a combination of multiple genetic variants, each contributing a small effect in conjunction with environmental and psychological factors [26,30].

To overcome the limitations of single-gene studies, genome-wide association studies (GWASs) have emerged as a powerful approach for identifying novel genetic variants associated with exercise addiction. Unlike single-gene analyses, GWASs simultaneously analyze thousands to millions of genetic variants across the entire genome. This comprehensive method enables researchers to uncover not only specific genes but also polygenic risk scores and pathways that may contribute to the development of exercise addiction [31,32,33]. For example, GWASs have successfully identified the genes related to dopamine regulation, stress response, and neuroplasticity, all of which may play a role in compulsive exercise behaviors.

Despite the potential of GWASs, the application of this method to exercise addiction remains sparse. The existing studies are limited in scope, and often characterized by small sample sizes and a lack of diversity in athletic populations and cultural contexts. This highlights the need for further GWAS research considering diverse sporting contexts, gender differences, and population-specific genetic characteristics. Such research could provide a more comprehensive understanding of the genetic and environmental interplay underlying exercise addiction, paving the way for targeted prevention and intervention strategies tailored to individual genetic profiles.

Considering these limitations and opportunities, the present study aimed to perform a GWAS of exercise addiction among elite Turkish wrestlers. This study systematically screened thousands of genetic variants across the participants’ genomes by conducting a DNA microarray-based strategy. Given the significant implications of exercise addiction for athletic performance and well-being, and the limited availability of genetic data on this phenotype, the present study sought to identify the genetic variants associated with exercise addiction profiles. Ultimately, it aimed to contribute to the growing body of knowledge in exercise and sports genetics, providing new insights into the biological mechanisms underlying this complex behavioral condition.

## 2. Materials and Methods

### 2.1. Ethical Approval

The present study was approved by the Clinical Research Ethics Committee of Gazi University (approval number 642, dated July 2023). The study adhered to the principles of the Declaration of Helsinki (World Medical Association, 2013; [34]) and the ethical standards of sports and exercise sciences. Written and verbal consent was obtained from each wrestler who participated in the study.

### 2.2. Participants

The sample comprised 67 male wrestlers affiliated with the Turkish Wrestling Federation. More specifically, the participants included 34 freestyle wrestlers (mean age = 23.2 years [SD = 5.3]; height = 175.8 cm [SD = 6.9]; weight = 80.1 kg [SD = 15.0]; sports experience = 12.5 years [SD = 4.2]) and 33 Greco-Roman wrestlers (mean age = 23.5 years [SD = 4.9]; height = 175.5 cm [SD = 7.7]; weight = 77.3 kg [SD = 14.5]; sports experience = 12.4 years [SD = 5.0]). All the participants were top-level national wrestlers who had competed in international events such as the Olympic Games, European Championships, and Balkan Championships. None of the athletes had tested positive for doping, and all were of Caucasian origin.

### 2.3. Measurements

#### 2.3.1. Exercise Addiction Scale (EAS)

The 17-item Exercise Addiction Scale [35] was used to assess exercise addiction. The scale comprises three factors: (i) excessive focusing and emotional distress (items 1–7), (ii) postponement and conflict of individual and social necessities (items 8–13), and (iii) tolerance and passion (items 14–17). The items are rated on a five-point Likert scale from 1 (strongly disagree) to 5 (strongly agree). The participants’ scores were categorized as follows: normal: 1–17; low-risk: 18–34; moderate-risk: 35–51; addicted: 52–69; and highly addicted: 70–85. In the present study, Cronbach’s alpha reliability coefficient was 0.76. Statistical analyses were conducted to compare exercise addiction levels across these categories, alongside genetic data, to investigate the potential associations between specific genetic variants and variability in the EAS scores.

#### 2.3.2. Genotyping

Genotyping was performed using the DNA extracted from 4 mL of peripheral venous blood collected from each participant. DNA isolation was carried out with the QIAamp Blood Mini Kit (Qiagen, Zug, Switzerland) following the manufacturer’s protocol. The Infinium Global Screening Array (Illumina Inc., San Diego, CA, USA) was employed to genotype more than 600,000 variants for each participant.

##### Genotype Quality Control

A total of 604,625 variants from 67 individuals were subjected to genotype quality control (QC). The selected QC filters were as follows:

1. Non-autosomal chromosomes were discarded.
2. Variants with gross genotyping errors or those highly deviating from Hardy–Weinberg Equilibrium (*p* < 1.0 × 10^−13^) were pruned.
3. The minor allele frequency (MAF) threshold was set at 0.01.
4. The genotype call rate threshold was set at a minimum of 0.90.

Consequently, 424,646 SNPs passed the selected criteria and were retained for further analyses.

### 2.4. Statistical Analysis

Fisher’s Exact Test was used for genome-wide association (2 × 2) analysis to investigate the potential associations with exercise addiction among wrestlers. After quality control, 424,646 SNPs were included in the analysis, with a genome-wide significance threshold set at 1.2 × 10^−^⁷ and a suggestive significance threshold set at 1.0 × 10^−^⁵ [36]. Manhattan plots were generated using CMplot in R version 4.4.1. [37], and statistical analyses were conducted using PLINK 1.9 [38]. For the analyses of the targeted SNPs, Fisher’s Exact Tests were conducted (*p* < 0.05).

## 3. Results

### 3.1. Exercise Addiction Scale

The participants were initially classified into five groups based on the EAS scores: normal, low-risk, risky, addicted, and highly addicted. However, due to small or zero sample sizes in some groups, the participants were reclassified into two categories: low-addiction risk (normal, low-risk, and moderate-risk) and high-addiction risk (addicted and highly addicted) (Table 1). Subsequently, addiction status was analyzed within each wrestling discipline (i.e., Greco-Roman and freestyle).

### 3.2. Genome-Wide Association Study

Among the Greco-Roman wrestlers, suggestive (*p* < 1 × 10^−^⁵) differences were observed in the frequencies of the *PRDM10* gene rs74345126 polymorphism and the intergenic variant rs72652685 (near the *PTPRU* gene) between the low- and high-addiction groups (Table 2). Among the freestyle wrestlers, the intergenic variant rs830747 (near the *EPHA4* gene) exhibited suggestive variation between the low-risk and high-addiction phenotypes (Table 2).

**Figure 1:**
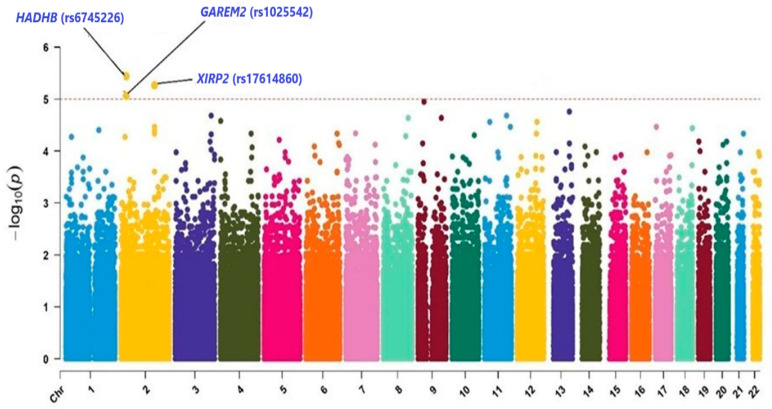
Manhattan plot based on the *p*-values of each SNP, analyzed in a sport-branch-independent and addiction-profile-dependent manner. Each color represents a chromosome, and each point corresponds to a single SNP. The horizontal line represents the suggestive significance threshold (*p* < 1.0 × 10^−5^), indicating the *p*-value cutoff used to identify potentially significant SNPs.

Further analysis combined the two groups of wrestlers to assess SNP associations with exercise addiction profiles. According to the Manhattan plot (Figure 1), significant differences were observed in the variants rs6745226 (*HADHB* gene), rs1025542 (near the *GAREM2* gene), and rs17614860 (*XIRP2* gene) between the low-risk and high-addiction groups. The allelic frequencies of these variants are presented in Table 3.

### 3.3. Candidate Gene Association Study

We also examined the potential associations between the genetic markers previously linked to addiction-related traits such as obsessive–compulsive disorder, cigarette smoking, and alcohol consumption, and personality traits linked to negative emotions including neuroticism. Using this approach, we identified three additional SNPs (*DEFB135* rs4841662, *BCL11A* rs7599488, and *CSRNP3* rs1551336) associated with exercise addiction (*p* < 0.05; Table 4).

## 4. Discussion

The present study explored the potential associations between genetic variants and exercise addiction among elite wrestlers using a GWAS approach. This methodology allows for the simultaneous analysis of thousands of genetic variants across the genome, offering a comprehensive understanding of the genetic architecture underlying complex phenotypes such as exercise addiction. To the best of the authors’ knowledge, this study is the first in the existing literature to employ GWAS for analyzing the exercise addiction phenotype. By focusing on elite athletes, this study also provided a unique perspective on how genetic predispositions interact with the high-intensity training demand characteristics of competitive sports.

Exercise addiction is closely associated with a complex interplay of psychological and behavioral factors that not only contribute to the onset of the condition but also exacerbate its severity. Among these, depressive symptoms stand out as a significant correlate, with research indicating that individuals may engage in excessive exercise as a maladaptive coping mechanism to manage underlying mood disturbances or feelings of low self-worth. This reliance on exercise for emotional regulation can create a cycle where temporary relief from depressive symptoms reinforces compulsive exercise behaviors, ultimately deepening the addiction [42].

Another critical factor is narcissistic tendencies, which often manifest among individuals who use exercise to enhance their appearance or achieve notable physical accomplishments. For such individuals, the validation of their self-worth becomes tied to external achievements or the admiration of others. This external focus can drive them to push beyond healthy limits, prioritizing appearance and performance over physical and psychological well-being [43]. Body image anxiety is another prevalent factor associated with exercise addiction, particularly among individuals who perceive themselves as falling short of the social or personal standards of physical appearance. This anxiety fuels a compulsive desire to achieve an ideal body, often leading to excessive and rigid exercise routines. Coupled with this is alexithymia, a condition characterized by difficulty in identifying and expressing emotions. Many individuals with exercise addiction struggle with emotional awareness, making it challenging for them to address their underlying psychological needs. This emotional disconnect can lead to the use of exercise as a substitute for addressing unprocessed emotions or unresolved conflicts, further entrenching the addiction [44].

Another significant factor is body dissatisfaction, where individuals excessively exercise to alter or maintain their physique, driven by unrealistic ideals or social pressures [45]. Exercise addiction often coexists with comorbid conditions, such as food addiction and eating disorders, where compulsive behaviors around food and exercise become intertwined [46]. Additionally, behaviors such as compulsive shopping [47] and psychological rigidity [48] reflect a pattern of maladaptive coping strategies and inflexible thinking, further complicating the psychological profile of affected individuals. These associations underscore that individuals with exercise addiction are not merely focused on physical activity but may experience a spectrum of maladaptive emotional, cognitive, and behavioral tendencies. This interconnectedness of psychological challenges often exacerbates their condition, making early identification and comprehensive intervention crucial.

The participants in the study were categorized into five addiction-related phenotypes: normal, low-risk, moderate-risk, addicted, and highly addicted. This classification was based on the scores obtained from the EAS [35], enabling a nuanced understanding of the spectrum of exercise addiction among the participants. Notably, the number of participants in the addicted and highly addicted groups was higher compared to other categories (Table 1). This distribution was expected, because elite athletes are required to train frequently and intensively, often leading to patterns of behavior that resemble addiction.

Additionally, significant differences were observed between the wrestling disciplines, with more freestyle wrestlers classified as highly addicted (n = 11) compared to Greco-Roman wrestlers (n = 3). This discrepancy may reflect the distinct training strategies employed in the two disciplines. Greco-Roman wrestlers primarily focus on building physical strength and power whereas freestyle wrestlers prioritize flexibility, which involves the ability to move muscles and joints through a full normal range of motion [49]. Although no direct correlation has been established between flexibility and exercise addiction, the diverse training modalities may shape the addiction profiles differently across disciplines [50]. These findings highlight the importance of considering sport-specific factors when analyzing exercise addiction.

At the molecular level of exercise addiction, the mechanisms particularly influencing the mesolimbic reward system have also been proposed as underlying contributors to addiction [51]. This system, which plays a crucial role in processing reward and motivation, may be dysregulated among individuals prone to compulsive behaviors. Supporting this notion, Cetin et al. [52] observed that athletes with higher levels of exercise addiction exhibited poorer athletic performance, emphasizing the negative impact of addiction on both mental health and physical performance. These findings underscore the need for an integrative approach to understanding exercise addiction, combining psychological, behavioral, and genetic perspectives.

A systematic review found that the risk of exercise addiction is higher among athletes compared to non-athletes, fueling ongoing debates about the definition and conceptualization of this condition [53]. Researchers have proposed two distinct categories of exercise addiction: primary and secondary. Primary exercise addiction is characterized by an intrinsic dependency on exercise, driven by motivations such as mastery, personal achievement, or the pursuit of performance improvement. In contrast, secondary exercise addiction arises when exercise is used to achieve external goals such as enhancing body image or managing eating disorders. Athletes are more likely to experience primary exercise addiction because their goals often revolve around improving performance and pushing their physical limits [54]. This distinction is critical for tailoring interventions because primary addiction may require strategies focused on intrinsic motivation and self-regulation whereas secondary addiction may necessitate addressing the underlying psychological or physical health issues.

In the genetic association approaches, the present study identified six SNPs suggestively associated with exercise addiction. These SNPs were the PR/SET Domain 10 (*PRDM10*) rs74345126, near Protein Tyrosine Phosphatase Receptor Type U (*PTPRU*) rs72652685, the Hydroxyacyl-CoA Dehydrogenase Trifunctional Multienzyme Complex Subunit Beta (*HADHB*) rs6745226, the Xin Actin Binding Repeat Containing 2 (*XIRP2*) rs17614860, near GRB2 Associated Regulator of MAPK1 Subtype 2 (*GAREM2*) rs1025542 and near EPH Receptor A4 (*EPHA4*) rs830747. Five of these variants have previously been linked to increased risks of mental health disorders such as anxiety and depression, or higher levels of physical activity. For *PRDM10* rs74345126, the G allele has been associated with increased risks of anxiety [55] and severe depression [56]. The *PRDM10* gene encodes a transcriptional regulator implicated in neuronal development and function processes [57] that are highly relevant to mental health and compulsive behaviors.

For rs72652685, located close to the *PTPRU* gene, the A allele is linked to a higher risk of mental health and behavioral disorders related to stimulant use [58]. The gene encodes the protein tyrosine phosphatase involved in cellular signaling and neural development [59], suggesting its potential role in addiction pathways. For *HADHB* rs6745226, the A allele is also associated with increased risks of anxiety and depression [55]. The *HADHB* gene encodes a mitochondrial enzyme involved in fatty acid beta-oxidation [60]. Mitochondrial dysfunction has increasingly been recognized as a contributing factor to neuropsychiatric disorders, potentially linking this gene to exercise addiction. For *XIRP2* rs17614860, the A allele has been associated with higher levels of physical activity [55]. The *XIRP2* gene encodes a protein that maintains sarcomere integrity, particularly in type IIX muscle fibers, suggesting its involvement in physical activity and potentially in exercise addiction [61].

For rs1025542, located close to the *GAREM2* gene, the G allele has been linked to increased risks of anxiety and depression [55]. The gene encodes a signaling adaptor protein involved in cellular stress responses and behavior [62]. The findings of the present study support the notion of shared genetic mechanisms underlying exercise addiction, mental health disorders, and high physical activity levels. These overlapping biological pathways suggest that the same genetic variants may influence multiple phenotypes such as addiction susceptibility, mental health status, and exercise performance. For instance, the genes involved in dopamine signaling, neuronal development, and mitochondrial function may simultaneously affect addiction-related behaviors and psychological well-being.

In addition to the GWAS approach, we also screened candidate SNPs relating to the emotional alterations that may be linked to the addiction profile. More specifically, it was found that *DEFB135* rs4841662 (a marker for neuroticism), *BCL11A* rs7599488 (a marker for smoking frequency), and *CSRNP3* rs1551336 (a marker for obsessive–compulsive disorder) were associated with exercise addiction. Notably, the BCL11 Transcription Factor A (*BCL11A*) rs7599488 A allele, previously identified as a risk allele for smoking frequency, was significantly associated with exercise addiction in both the wrestling disciplines and even more strongly among the combined cohort of wrestlers (*p* = 0.001). This finding suggests that *BCL11A* rs7599488 may serve as a robust genetic marker for exercise addiction. The Defensin Beta 135 (*DEFB135*) gene encodes a beta-defensin, a type of antimicrobial peptide involved in regulating immune responses and inflammation. The dysregulation of immune and inflammatory pathways has been increasingly recognized as a factor influencing neuropsychiatric traits, such as neuroticism [63], potentially linking this gene to exercise addiction.

The *BCL11A* gene encodes a transcription factor essential for brain development [64], particularly in the modulation of dopaminergic signaling pathways [65] that underlie reward processing and addiction mechanisms. The A allele of *BCL11A* rs7599488, previously associated with increased smoking frequency [40], may similarly heighten vulnerability to exercise addiction by enhancing reward-seeking behaviors. The Cysteine-Serine-Rich Nuclear Protein 3 (*CSRNP3*) gene encodes a transcriptional regulator involved in the positive regulation of apoptotic processes and transcription mediated by RNA polymerase II. Through these roles, it may influence various functions. Although more research is needed, it can be hypothesized that the dysregulation of these stress-response pathways may contribute to compulsive exercise behaviors, aligning with the genetic basis of obsessive–compulsive disorder [41].

The present study demonstrated several strengths in its exploration of exercise addiction’s genetic underpinnings among elite wrestlers. One major strength lies in its novelty because it broadens the scope of addiction research to include a specific athletic population. The categorization of the participants into five distinct addiction-related phenotypes provided a granular understanding of the spectrum of exercise addiction. Moreover, the study contextualized its findings within the specific training modalities of freestyle and Greco-Roman wrestling, offering discipline-specific insights that enhance the relevance of the results.

However, the study has several limitations that warrant consideration. The small sample size limits the generalizability of the findings to broader populations, and the reduced statistical power associated with sub-sample analyses for freestyle and Greco-Roman wrestlers increases the risk of false negatives. The multiple testing required for SNP associations also heightens the potential for type I errors, necessitating cautious interpretation and independent replication. Additionally, the study was unable to establish mechanistic links or causality between the identified SNPs and exercise addiction, because the observed genetic associations with psychiatric conditions were correlational and may oversimplify the complex biological pathways involved. The reliance on prior associations of SNPs (e.g., in or near *PRDM10*, *PTPRU*, *HADHB*, *XIRP2*, and *GAREM2*) identified in distinct populations, trait definitions, or disease phenotypes further limits the ability to directly extrapolate these findings to exercise addiction, especially in the absence of biochemical or gene expression validation. Moreover, the study’s exclusive focus on elite athletes, who engaged in frequent and intense training, may skew the results, because this population is predisposed to higher exercise addiction scores on the EAS. The lack of systematic control for behavioral and environmental factors, such as training regimens, coaching styles, dietary habits, and psychosocial stress, could also confound the observed genetic associations.

Replicating these findings among larger, more diverse populations, including non-athletes and athletes from other sports, is essential to confirm the robustness of the identified genetic associations and their broader applicability. Future research should expand upon these findings by incorporating larger and more varied samples, investigating the genetic underpinnings of exercise addiction across different sports and recreational exercise contexts. Integrating GWAS findings with advanced techniques, such as epigenetics and transcriptomics, could provide further insights into the molecular mechanisms underlying exercise addiction. From a practical perspective, raising awareness about exercise addiction among athletes, coaches, and healthcare professionals is crucial. Recognizing the signs of addiction and understanding its genetic and psychological contributors can inform the development of targeted interventions that prioritize a balanced approach to exercise and enhance performance while safeguarding mental and physical health.

## 5. Conclusions

The present study provides preliminary evidence for the genetic basis of exercise addiction, highlighting eight SNPs (*PRDM10* rs74345126, near *PTPRU* rs72652685, *HADHB* rs6745226, *XIRP2* rs17614860, near *GAREM2* rs1025542, *DEFB135* rs4841662, *BCL11A* rs7599488, and *CSRNP3* rs1551336) that may play a role in the development of this condition among elite wrestlers. These findings underscore the need for further research to confirm these associations and explore the underlying mechanisms, which could inform the development of targeted interventions for exercise addiction. In the future, it will be essential to investigate the hypothesis that specific genomic predictors of physical activity might be associated with exercise addiction [66].

## Figures and Tables

**Table 1:** Prevalence of wrestlers across different addiction categories.

Addiction Profile	Total N	Freestyle N	Freestyle Mean Score	Greko-Roman N	Greko-Roman Mean Score
Normal	0	0	0	0	0
Low-risk	1	1	34	0	0
Moderate-risk	9	4	44.75	5	46.6
Addicted	43	18	59.42	25	60.64
Highly addicted	14	11	72.33	3	71.33

**Table 2:** Most significant genetic variants associated with exercise addiction among the two groups of wrestlers.

Greko-Roman Addiction Level Comparison				
Genetic Variants	Low-Addiction Risk AF	High-Addiction Risk AF	*p*-Value	OR
*PRDM10* rs74345126	A allele: 60% G allele: 40%	A allele: 0% G allele: 100%	2.3 × 10^−6^	0.000
Near *PTPRU* rs72652685	C allele: 80% A allele: 20%	C allele: 9% A allele: 91%	8.6 × 10^−6^	0.025
Freestyle Addiction Level Comparison				
Near *EPHA4* rs830747	G allele: 90% A allele: 10%	G allele: 15% A allele: 85%	8.5 × 10^−6^	0.02

Suggestively (*p* < 1 × 10^−^⁵) associated SNPs are shown. AF, allelic frequency. OR, odds ratio.

**Table 3:** Most significant genetic variants associated with exercise addiction in the combined cohort of wrestlers.

Genetic Variants	Low-Addiction Risk AF	High-Addiction Risk AF	*p*-Value	OR
*HADHB* rs6745226	C allele: 55% A allele: 45%	C allele: 7.9% A allele: 92.1%	3.6 × 10^−6^	0.070
*XIRP2* rs17614860	G allele: 30% A allele: 70%	G allele: 0% A allele: 100%	5.4 × 10^−6^	0.000
Near *GAREM2* rs1025542	A allele: 65% G allele: 35%	A allele: 15% G allele: 85%	8.6 × 10^−6^	0.094

Suggestively (*p* < 1 × 10^−^⁵) associated SNPs are shown. AF, allelic frequency. OR, odds ratio.

**Table 4:** Genetic variants associated with exercise addiction in different cohorts of wrestlers, overlapping with SNPs previously reported to be linked to addiction-related traits and negative emotions.

Risk Allele for Both Traits	Trait from the Literature	*p*-Value for the Trait	Reference	Athletic Cohort	*p*-Value for the Exercise Addiction
*DEFB135* rs4841662 G	Neuroticism	1.6 × 10^−21^	[39]	Freestyle wrestlers	0.035 *
*BCL11A* rs7599488 A	Cigarettes per day	9.0 × 10^−9^	[40]	Freestyle wrestlers	0.039 *
				Greko-Roman wrestlers	0.015 *
				All wrestlers	0.001 *
*CSRNP3* rs1551336 C	Obsessive–compulsive disorder	2.2 × 10^−8^	[41]	Greko-Roman wrestlers	0.015 *

* *p* < 0.05, statistically significant association with exercise addiction.

## Data Availability

The data presented in this study are available upon request from the first author, as data in genetic research is sometimes kept confidential for ethical reasons.
